# Effects of JWA, XRCC1 and BRCA1 mRNA expression on molecular staging for personalized therapy in patients with advanced esophageal squamous cell carcinoma

**DOI:** 10.1186/s12885-015-1364-0

**Published:** 2015-04-30

**Authors:** Bin Wei, Qin Han, Lijuan Xu, Xiaohui Zhang, Jing Zhu, Li Wan, Yan Jin, Zhaoye Qian, Jingjing Wu, Yong Gao, Jianwei Zhou, Xiaofei Chen

**Affiliations:** 1 Department of Medical Oncology, Huai’an First People’s Hospital, Nanjing Medical University, Huai’an, 223300 China; 2 Department of Molecular Cell Biology and Toxicology, Jiangsu Key Lab of Cancer Biomarkers, Prevention & Treatment Cancer Center; School of Public Health, Nanjing Medical University, Nanjing, 210029 China

## Abstract

**Background:**

DNA damage repair genes JWA, XRCC1 and BRCA1 were associated with clinical outcomes and could convert the response to the cisplatin-based therapy in some carcinomas. The synergistic effects of JWA, XRCC1 and BRCA1 mRNA expression on personalized therapy remain unknown in advanced esophageal squamous cell carcinoma (ESCC).

**Methods:**

We employed quantitative real-time polymerase chain reaction (qPCR) to determine the expression of JWA, XRCC1 and BRCA1 mRNA in paraffin-embedded specimen from 172 patients with advanced ESCC who underwent the first-line cisplatin-or docetaxel-based treatments.

**Results:**

High JWA or XRCC1mRNA expression was correlated with longer median overall survival (mOS) in all the patients (both *P* < 0.001) or in subgroups with different regimens (all *P* < 0.05), but not correlated with response rate (RR, all *P* > 0.05). Multivariate analysis revealed that high JWA (HR 0.22; 95% CI 0.13-0.37; *P* < 0.001) or XRCC1 (HR 0.36; 95% CI 0.21-0.63; *P* < 0.001) mRNA expression emerged as the independent prognostic factors for ESCC patients in this cohort. But no significant difference in prognostic efficacy was found between JWA plus XRCC1 and JWA alone through ROC analysis. Further subgroup analysis showed cisplatin-based treatments could improve mOS of patients with low JWA expression (*P* < 0.05), especially in those with low BRCA1 expression simultaneously (*P* < 0.001); while in patients with high JWA expression, high BRCA1 mRNA expression was correlated with increased mOS in docetaxel-based treatments (*P* = 0.044).

**Conclusion:**

JWA, XRCC1and BRCA1 mRNA expression could be used as predictive markers in molecular staging for personalized therapy in patients with advanced ESCC who received first-line cisplatin- or docetaxel-based treatments.

**Electronic supplementary material:**

The online version of this article (doi:10.1186/s12885-015-1364-0) contains supplementary material, which is available to authorized users.

## Background

Esophageal cancer was the eighth most common cancer with rapidly increasing incidence and the sixth leading cause of cancer-related death worldwide as estimated in 2008 [1]. Despite the progress in the multimodal therapy with the combination of operation, radiotherapy and chemotherapy, the overall 5-year survival rate of this disease only ranged from 15% to 25% [2,3]. Tailored treatment based on molecular staging might help to determine the optimal regimen for the right patients on the right time, which would contribute to improving clinical outcomes and limiting toxic and side effects [4]. Although some molecular markers have been identified for personalized treatment of esophageal cancer, such as cisplatin related markers [breast cancer susceptibility gene 1 (BRCA1) and excision repair cross-complementing 1 (ERCC1)], 5-FU related markers [dihydropyrimidine dehydrogenase (DPD) and thymidylatesynthase (TS)] and docetaxel related markers (BRCA1) [5-7], the molecular backgrounds remain largely unclear to determine therapeutic effectiveness in esophageal cancer systematically.

In our previous researches, JWA was identified as a novel microtubule-associated gene which encoded the ADP-ribosylation-like factor 6 interacting protein 5 (ARL6ip5) involved in cell oxidative stress, differentiation and apoptosis [8-10]. Also, JWA was found to regulate cancer cells migration via MAPK cascades and suppress the ability of adhesion, invasion and metastasis by integrin alphaVbeta3 signaling [11,12]. Recently, we confirmed that JWA was a base excision repair (BER) protein which could regulate X-ray repair cross complement group 1(XRCC1) and participated in the DNA damage repair pathway through stabilizing BER protein complex to facilitate the repair of DNA single-strand breaks (SSB) [13]. The DNA repair protein XRCC1 interacted with enzymatic components which include PARP-1, DNA polymerase β, APE1, PNK, PCNA and DNA ligase III in BER pathway [14-19]. Single nucleotide polymorphisms (SNPs) of XRCC1 gene acted as a risk factor might be a valuable genetic marker for chemotherapy in various cancers containing esophageal cancer [20-22]. Our group has demonstrated the possibility of JWA participating in tailored therapy of tumor for its novel function as regulator of XRCC1. In previous clinical studies, we confirmed that the expressions of JWA and XRCC1 protein were significant prognostic and predictive biomarker in hepatocellular carcinoma and gastric cancer [23-25]. Also, the other DNA repair proteinBRCA1 which was involved in nucleotide excision repair (NER) and DNA double single break repair (DSBR) pathway was found to participate in the inverse resistance to cisplatin- or docetaxel-based treatments in patients with esophageal cancer [7,26].

However, whether combination of JWA, XRCC1 and BRCA1 mRNA expressions could be used as prognostic markers in molecular staging for tailored therapy in ESCC needs to be determined in clinic urgently. In present study, the aim is to investigate the prognostic and predictive roles of JWA/XRCC1 mRNA expressions and to explore the synergistic effect of JWA/XRCC1/BRCA1mRNA expression on molecular staging in personalized therapy of advanced ESCC who received cisplatin- or docetaxel-based treatments.

## Methods

### Patients

A total of 172 patients enrolled in the study were all histologically confirmed to be locally advanced or metastatic ESCC (stage II-IV) and had available paraffin-embedded tumor material for molecular analysis. They all had measurable lesions with a better Eastern Cooperative Oncology Group performance status (PS; 0 to 2). Among them, 81 patients with metastatic or with surgically unresectable disease who couldn’t tolerate radiotherapy underwent cisplatin- or docetaxel-based chemotherapy as the first-line treatment. The chemotherapy regimens included cisplatin-based regimens (cisplatin 25 mg/m^2^ on day 1-3 plus 5-fluorouracil 500 mg/m^2^ on day 1-5) and docetaxel-based regimens (docetaxel 60-75 mg/m^2^ plus 5-fluorouracil 500 mg/m2 on day 1-5). Chemotherapy was repeated every 3-4 weeks for a maximum of six cycles unless patients had disease progression or in unsupportable adverse reactions. The other 91 patients with locally advanced disease underwent cisplatin or docetaxel-based concurrent chemoradiotherapy (CCRT) or radiotherapy alone as the first-line treatment. CCRT consisted of chemotherapy and concurrent thoracic radiotherapy. The chemotherapy regimens comprised weekly cisplatin (25 mg/ m^2^ on day 1 per week) plus 5-fluorouracil (300 mg/ m^2^ on day 1-3 per week) or docetaxel (25 mg/ m^2^ on day 1 per week) plus 5-fluorouracil (300 mg/m^2^ on day 1-3 per week) for 5 weeks. CT simulation and 3 D treatment planning were used in the concurrent thoracic radiotherapy with radiation dose of 50-60 grays (Gy) over 5 weeks (2 Gy/fraction per day, 5 fractions per week). Barium swallow and computed tomography scans were utilized in baseline and restaging assessment every 2 cycle of chemotherapy or 4 weeks after radiotherapy.

The institutional approval was obtained from ethics committee of Huai’an First Hospital of Nanjing Medical University and all patients signed their informed consent for the use of tissue material in this translational research.

### qPCR analysis for JWA, XRCC1 and BRCA1 mRNA expression

We assessed JWA, XRCC1 and BRCA1 mRNA expression in paraffin-embedded tumor specimens obtained by biopsy under endoscope from 172 patients as described in previous study [7]. Before RNA preparation, micro-dissection was performed to ensure serial sections of 7-mm thickness with more than 80% of tumor cells. The pellet of micro-dissected cells was resuspended in RNA lysis buffer supplemented with proteinase K after paraffin was removed by xylene. RNA was then extracted with phenol-chloroform-isoamyl alcohol and precipitated with isopropanol in the presence of glycogen and sodium acetate. Subsequently RNA was treated with DNase to avoid genomic DNA contamination. The M-MLV reverse transcriptase was used to synthesize cDNA. Template cDNA was amplified with specific primers for JWA, XRCC1, BRCA1 and β-actin as follows: 5′-GGAGGAGTCATTGTGGTGC-3′ (forward) and 5′-GAAGTCTCAGGGATGCGTG-3′ (reverse) for JWA; 5′-CTTTGTGGAGGTGCTAGTGG-3′ (forward) and 5′-ATGGCGAGTCCTTGCTGT-3′ (reverse) for XRCC1; BRCA1 5′-GGCTATCCTCTCAGAGTGACATTTTA-3′ (forward) and 5′-GCTTTATCAGGTTATGTTGCATGGT-3′ (reverse) for BRCA1, 5′-TGAGCGCGGCTACAGCTT -3′ (forward) and 5′-TCCTTAATGTCACGCACGATTT -3′ (reverse) for β-actin. Gene expression was quantified by quantitative real-time polymerase chain reaction (qPCR) through the 7900HT Sequence Detection System (Applied Biosystems, Foster City, CA) and the qPCR products were detected by fluorochrome dye SYBR Premix Ex TaqTM (TaKaRa, Japan). Each sample was assayed in triplicate with RNase-free water as negative control and commercial RNA as positive control. Relative gene expression quantification was conducted according to the comparative quantification cycle (Cq) method using β-actin as an endogenous control and commercial RNA controls (human lung and liver RNA; Strata gene, La Jolla, CA, USA) as calibrators. In all experiments, only triplicates with a standard deviation (SD) of the Cq value < 0.30 were accepted. Final values were determined by the formula 2^-△△Cq^ [=2^- (Cq sample - Cq calibrator)^]. All experiments were conducted in the Department of Molecular Cell Biology and Toxicology, Nanjing Medical University (Nanjing, China).

### Study design and statistical analysis

The primary endpoint of this study was to examine the potential prognostic and predictive roles of JWA/XRCC1 mRNA expressions and to explore the synergistic effect of JWA/XRCC1/BRCA1mRNA expression on overall survival and clinical responses in ESCC patients treated with cisplatin- or docetaxel-based regimens in the first-line. Overall survival was calculated from the date of pathologic diagnosis to the date of death or last follow-up or death from any cause. The clinical response was assessed according to the Response Evaluation Criteria Evaluation in Solid Tumors (RECIST) [27]. The Tumor Node Metastasis (TNM) system was used to classify the tumor stage. Progression-free survival was not examined because we could not get exact time of progress-free survival of the patients who did not receive further assessments of disease after fist-line treatment in the present retrospective study. The median value was employed as the cut-off points [7,28]. Samples with mRNA expression above the median were considered as high expression, whereas those with value below or equal to the median as low expression. The distributions of patients were reported with demographic, clinical and biological characteristics. The absolute frequencies and percentages were used to depict qualitative variables, and the median values and ranges were used to describe quantitative variables. The normality of quantitative variables was analysed by the Kolmogorov-Smirnov test and compared with the Mann-Whitney U test. The potential association between clinical characteristics, response and gene expression levels were compared with two-sided chi-square test or Fisher exact test. The distributions of OS were ascertained to probe for the significance by using Kaplan-Meier method and compared with the two-sided log-rank test. A multivariate Cox regression analysis with hazard ratios (HR) and 95% confidence intervals (95% CI) were used to assess the association between each potential prognostic factor and survival. The time-dependent ROC curve analysis for censored data and the area under the Curve (AUC) of the ROC curves were used to analyse the predictive value of the parameters [29]. Statistical significance was considered to be *P* ≤ 0.05. Statistical analyses were performed with the Statistical Package for the Social Sciences (SPSS) for Windows version 19.0 (SPSS Inc, Chicago, IL).

## Results

### Patients’ characteristics

Clinical data and paraffin-embedded samples from the primary tumors were collected from 172 ESCC patients treated with cisplatin- or docetaxel-based chemotherapy/chemoradiotherapy in our center. Successful amplification of three genes of JWA, XRCC1 and BRCA1 was achieved in 145 specimens. The median age was 62 (44-84 years) and 92 patients were male. The majority of patients had PS 0-1. Among them, 73 patients treated with chemotherapy had stage III–IV and other 72 patients treated with chemoradiotherapy or radiotherapy alone had stage II–III at the time of diagnosis. The clinical response rates (RR) were 68.5% and 83.3% in the two treatment groups, separately. After a median follow up period of 52.0 months (range 19.0-100.0), the mOS was 13.0 months (95% CI: 11.3-14.7) in chemotherapy group; while the mOS was 13.5 months (95% CI: 11.3-15.7) after a median follow-up period 48.0 months (range 20.0-100.0) in chemoradiotherapy group. All patient characteristics were shown in Table 1.

**Table 1:** **Patient characteristics of advanced (stage II–IV) esophageal cancer patients**

		Chemotherapy		Chemoradiotherapy		
Characteristics	All patients	Cis/ 5-Fu	Doc/ 5-Fu	Radio alone	Cis/ 5-Fu/ Radio	Doc/ 5-Fu/ Radio
Patients, No. (%)	145 (100)	35 (24.1)	38 (26.2)	12 (8.3)	30 (20.7)	30 (20.7)
Age, y median (range)	62 (44-84)	60 (48-81)	61 (45-78)	68 (44-83)	63 (45-74)	61 (52-84)
Sex (%)						
Males	92 (63.4)	25 (27.2)	24 (26.1)	6 (6.5)	19 (20.6)	18 (19.6)
Females	53 (36.6)	10 (18.9)	14 (26.4)	6 (11.3)	11 (20.8)	12 (22.6)
ECOG, PS (%)						
0-1	133 (91.7)	32 (24.1)	34 (25.6)	12 (9.0)	27 (20.3)	28 (21.0)
2	12 (8.3)	3 (25.0)	4 (33.3)	0 (0.0)	3 (25.0)	2 (16.7)
TNM stage (%)						
II	28 (19.3)	0 (0.0)	0 (0.0)	12 (42.9)	15 (53.6)	1 (3.5)
III	47 (32.4)	2 (4.2)	1 (2.1)	0 (0.0)	15 (31.9)	29 (61.7)
IV	70 (48.2)	33 (47.1)	37 (52.8)	0 (0.0)	0 (0.0)	0 (0.0)
G stage (%)						
G1	20 (13.8)	4 (20.0)	3 (15.0)	8 (40.0)	3 (15.0)	2 (10.0)
G2	90 (62.1)	25 (27.7)	24 (26.7)	3 (3.3)	24 (26.7)	14 (15.6)
G3	35 (24.1)	6 (17.1)	11 (31.4)	1 (2.9)	3 (8.6)	14 (40.0)
JWA median (range)	2.4(0.0002-126.0)	3(0.001-29.1)	2.2(0.0002-126.0)	3.9(0.3-67.8)	3.2(0.0014-50.1)	2.5(0.001-34.8)
XRCC1 median (range)	8.6(0.03-1410.4)	11.3(0.04-190.7)	5.5(0.03-211.7)	11.9(0.9-327.6)	9(0.1-1410.4)	7.3(0.03-64.3)
BRCA1 median (range)	11.4(0.4–70.0)	10.2(0.4–70.0)	10.9(0.6–44.9)	10.3(0.4–44.2)	11.4(1.2-62.9)	12.2(2.0–58.9)
Response rate (CR + PR), No. (%)		50 (68.5)		60 (83.3)		
Median OS (months, 95%CI)		13 (11.3-14.7)		13.5 (11.3-15.7)		

Cis = cisplatin; 5-Fu = 5-fluorouracil; Doc = docetaxel; ECOG = Eastern Cooperative Oncology Group; PS = Performance status; G = differentiation grade; CR = complete response; PR = partial response; OS = overall survival; CI = confidence interval; y = years.

### Genes’ mRNA expression levels and treatment outcomes

The median mRNA expression levels were 2.4 (range 0.0002-126.0) for JWA, 8.6(range 0.03-1410.4) for XRCC1 and 11.4 (range 0.4-70.0) for BRCA1. JWA mRNA expression was significantly associated with clinical features including patients’ gender and tumor differentiation grade. The JWA expression was higher in the females than the males (*P* = 0.025) and positively correlated with tumor differentiation grade stage (G stage, *P* = 0.047). There was no other association between clinical features and JWA, XRCC1 or BRCA1 mRNA levels (all *P* > 0.05) (Table 2). As to correlations of three genes, we observed the positive correlation between JWA and XRCC1 mRNA expression (Spearman’s test 0.67; *P* < 0.001) while BRCA1 was not correlated with JWA or XRCC1 expression (Spearman’s test 0.007, -0.131; *P* = 0.937, 0. 115).

**Table 2:** **Clinical characteristics associated with JWA, XRCC1 and BRCA1 mRNA expression levels**

	JWA level			XRCC1 level			BRCA1 level			Overall survival	
Characteristics	Low	High	*P* value	Low	High	*P* value	Low	High	*P* value	mOS (95%CI)	*P* value
Age, y											
≤62	39	36		40	35		37	38		13.0(11.3-14.8)	
>62	34	36	0.741	33	37	0.456	37	33	0.671	13.0(10.0-16.0)	0.805
Gender											
Males	53	39		52	40		47	45		12.0(10.1-13.9)	
Females	20	33	0.025	21	32	0.053	27	26	0.987	16.0(13.4-18.6)	0.007
ECOG, PS											
0-1	66	67		68	65		67	66		14.0(12.3-15.4)	
2	6	6	0.98	5	7	0.53	7	5	0.597	6.0(4.9-7.8)	<0.001
TNM stage											
II	12	16		14	14		18	10		14.0(11.9-16.1)	
III	25	22		25	22		22	25		10.0(8.3-11.7)	0.096
IV	36	34	0.666	34	36	0.886	34	36	0.290	14.0(11.4-14.6)	0.656
G stage											
G1	5	15		7	13		9	11		18.5(15.8-21.2)	
G2	48	42		47	43		50	40		13.0(11.4-14.7)	0.061
G3	20	15	0.047	19	16	0.328	15	20	0.375	9.0(6.7-11.3)	0.005

mOS = median overall survival; y = years; G = differentiation grade; ECOG = Eastern Cooperative Oncology Group; PS = performance status.

**Figure 1:**
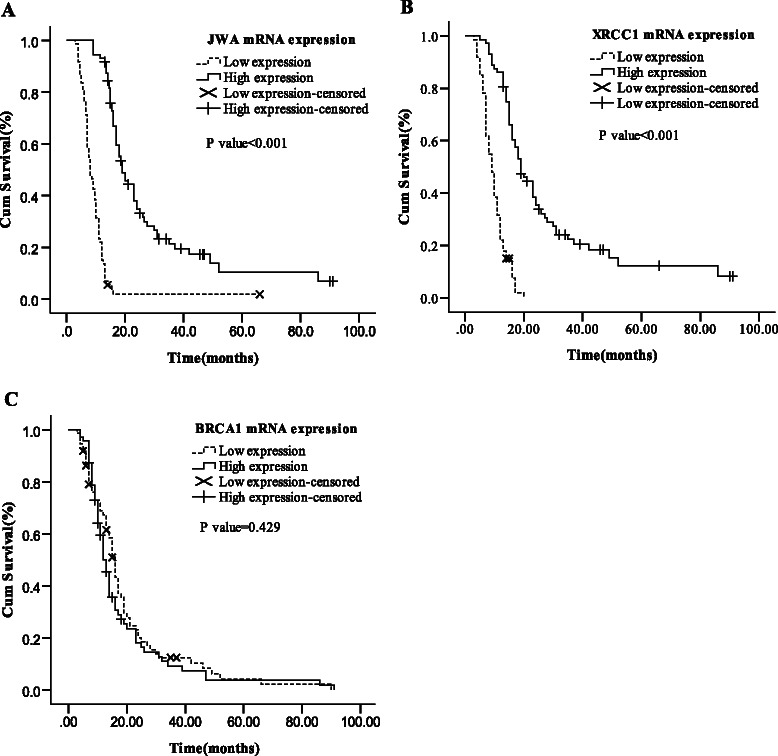
Median OS in total 145 advanced and metastasis esophageal cancer patients (stage II–IV) according to the mRNA expression levels. **(A)** JWA mRNA expression. **(B)** XRCC1 mRNA expression. **(C)** BRCA1 mRNA expression.

Clinical outcomes according to JWA, XRCC1 and BRCA1 mRNA expression were shown in Table 3. In the whole cohort, patients with high JWA mRNA expression had an increased median overall survival (mOS, 19.0 vs. 8.0 months, *P* < 0.001, Figure 1A) compared with those with low JWA expression. Similarly, patients with high XRCC1 mRNA expression also experienced longer mOS (19.0 *vs.* 9.0 months, *P* < 0.001; Figure 1B) in comparison with those with low XRCC1 expression. Conversely, no significant difference was observed in mOS (15.0 *vs.* 14.0 months, *P* = 0.429; Figure 1C) of patients according to BRCA1 expression levels. Moreover, there was no difference in term of clinical RR according to the expression of JWA (83.3% *vs.* 68.5%, *P* = 0.052), XRCC1 (83.3% *vs.* 68.5%, *P* = 0.052) and BRCA1 (78.7% *vs.* 71.8%, *P* = 0.267).

**Table 3:** **Treatment outcomes according to genes expression levels**

			RR, N (%)			mOS (months)	
Gene	Level	No.	CR + PR	SD + PD	*P* value	Median (95% CI)	*P* value
JWA	Low	73	68.5	31.5		8.0(6.6-9.4)	
	High	72	83.3	16.7	0.052	19.0(15.8-22.2)	<0.001
XRCC1	Low	73	68.5	31.5		9.0(7.5-10.5)	
	High	72	83.3	16.7	0.052	19.0(15.4-22.6)	<0.001
BRCA1	Low	74	79.7	20.3		15.0(14.4-17.6)	
	High	71	71.8	28.2	0.267	14.0(11.5-14.5)	0.429

mOS = median overall survival; RR = response rate; CI = confidence interval; CR = complete response; PR = partial response; SD = stable disease; PD = progress disease.

**Figure 2:**
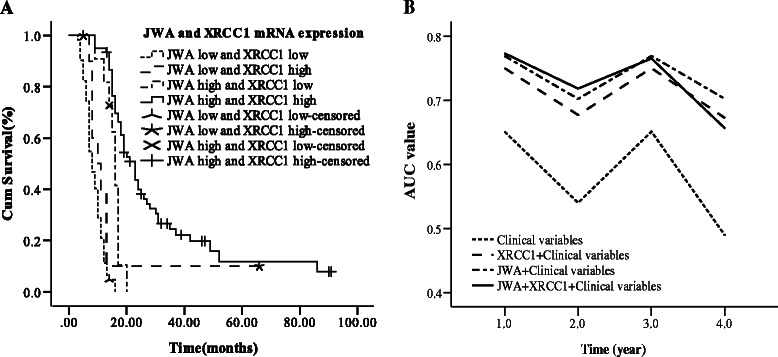
Combined effects of JWA/XRCC1 mRNA expression. **(A)** Median OS according to the combination of JWA/XRCC1 expression levels. **(B)** Time-dependent ROC analyses for the clinical risk score (TNM stage and G stage), combination of JWA or XRCC1.

To further assess the synergistic efficacy of JWA and XRCC1 mRNA expression on survival, the patients were then stratified into 4 distinct groups depending on gene expression of JWA and XRCC1: both high, JWA high/ XRCC1 low, JWA low/ XRCC1 high and both low. It was shown that patients with both high or JWA high/ XRCC1 low (mOS were 21.0 and 17.0 months) had a better outcome of survival than in the other 2 groups (mOS were 10.0 and 8.0 months), which indicated that JWA mRNA expression mainly affected overall survival of patients in this cohort (Figure 2A, Additional file 1: Table S1). To further evaluate the prognostic efficacy of JWA, time-dependent ROC analysis was carried out for the censored data. The combination of clinical risk score (TNM stage and G stage) and JWA or XRCC1 or JWA plus XRCC1 contributed much more than clinical risk score alone. However, JWA plus XRCC1 was not superior to JWA which contributed much more than XRCC1 (Figure 2B, Additional file 1: Table S2). For example, the AUC at year 3 was 0.765 for the combination of clinical risk score with JWA plus XRCC1 and 0.769 for the combination of clinical risk score with JWA, which showed that JWA plus XRCC1 mRNA expression were not better than JWA alone as a predictor for survival of patients with ESCC in present study.

### Treatment outcomes according to regimens or gene expression levels

**Figure 3:**
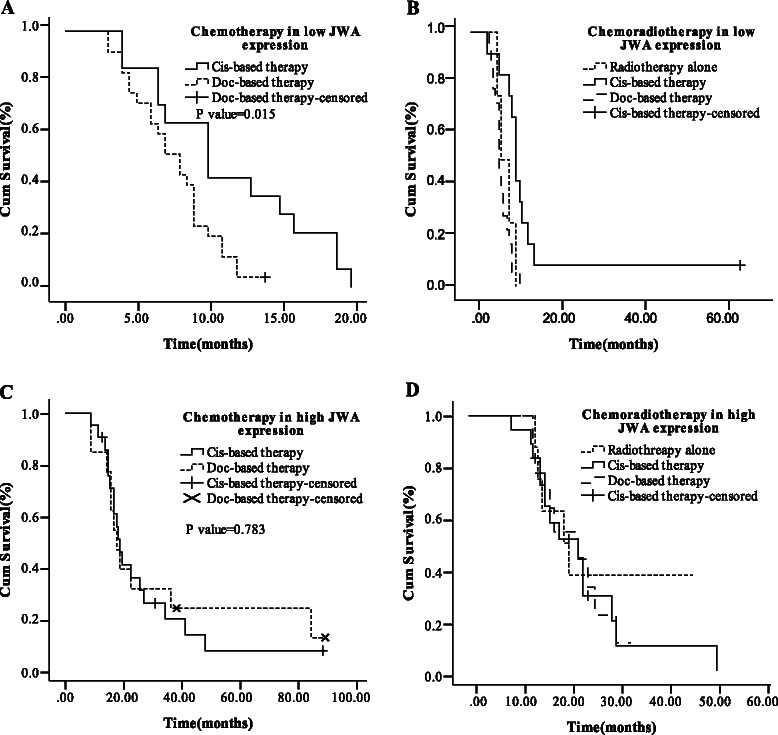
Median OS in low and high JWA expression levels according to different regimens. **(A)** Chemotherapy in low JWA expression. **(B)** Chemoradiotherapy in low JWA expression. **(C)** Chemotherapy in high JWA expression. **(D)** Chemoradiotherapy in high JWA expression.

In the further subgroup analysis stratified by regimens, high JWA or XRCC1 mRNA expression was all correlated with longer mOS (all *P* < 0.05) but not correlated with RR (all *P* > 0.05) in each subgroup with cisplatin- or docetaxel-based chemotherapy/chemoradiotherapy (Additional file 1: Table S3, S5 andAdditional file 2: Figure S1, S2). In the chemotherapy group stratified by gene expression, patients with low JWA mRNA expression had increased mOS when treated with cisplatin-based chemotherapy compared to docetaxel-based chemotherapy (10.0 *vs.* 8.0 months, *P* = 0.015; Figure 3A and Additional file 1: Table S4). In the chemoradiotherapy group, cisplatin-based chemoradiotherapy was the best choice of treatment for patients with low JWA expression compared with docetaxel-based chemoradiotherapy or radiotherapy alone [mOS were 11.0, 7.0 (*P* = 0.001) and 7.5 months (*P* = 0.537), respectively; Figure 3B and Additional file 1: Table S4]. However, no significant difference of mOS was found in patients with high JWA or low XRCC1 or high XRCC1 mRNA expression between cisplatin-based and docetaxel-based treatments (all *P* > 0.05; Figure 3C, D and Additional file 1: Table S4, S6, Additional file 2: Figure S3). Low BRCA1 mRNA expression correlated with increased mOS (*P* = 0.001 and *P* = 0.003, respectively) in cisplatin-based chemotherapy or chemoradiotherapy group and inversely correlated with deceased mOS (*P* = 0.020 and *P* = 0.048, respectively) in docetaxel-based chemotherapy or chemoradiotherapy group, which was in line with the results shown in the previous research [7] (Additional file 1: Table S7, S8 and Additional file 2: Figure S4, S5).

### Prognostic value of combining JWA with BRCA1 mRNA expression according to regimens

**Figure 4:**
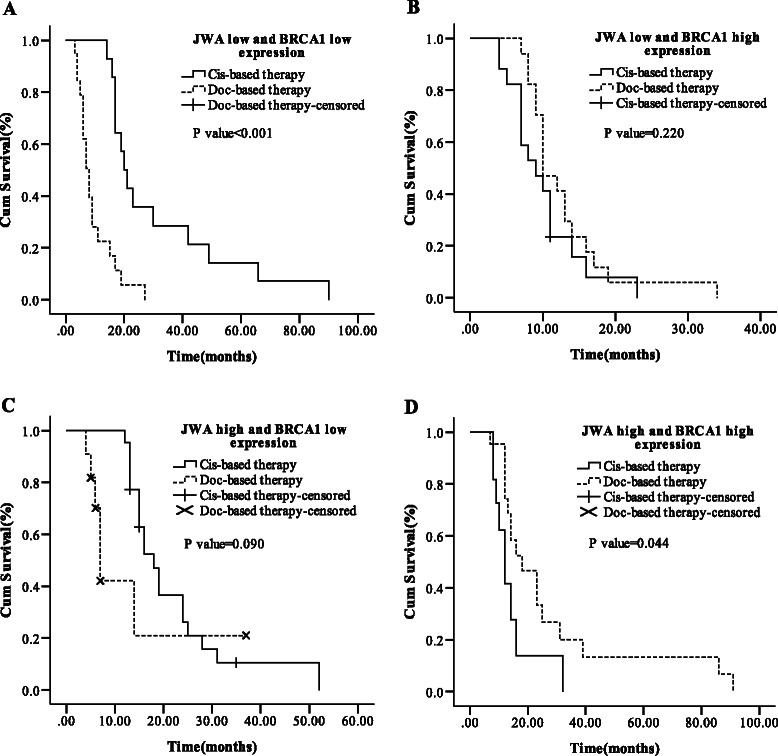
Median OS according to the cis-based or doc-based treatments in different groups. **(A)** Low JWA and low BRCA1 mRNA expression. **(B)** Low JWA and high BRCA1 mRNA expression. **(C)** High JWA and low BRCA1 mRNA expression. **(D)** High JWA and high BRCA1 mRNA expression.

For effects of JWA or BRCA1 mRNA expression on survival of ESCC in term with regimens, the prognostic value of combination with the two genes was further investigated in subgroup analysis according to treatments (Figure 4 and Additional file 1: Table S9). We found that cisplatin-based treatment brought a better survival (20.0 *vs.* 8.0 months; *P* < 0.001) to patients with low JWA/ low BRCA1 mRNA expression, whereas docetaxel-based treatment prolonged survival of those with high JWA/high BRCA1 mRNA expression (18.0 *vs.* 12.0 months; *P* = 0.044). No significant difference of survival was observed in the subgroups with one gene high (JWA or BRCA1 high) between cisplatin-based and docetaxel-based treatments (all *P* > 0.050).

### Univariate and multivariate analyses

Univariate analysis demonstrated that significant association was observed between mOS and PS (*P* < 0.001), gender (*P* = 0.007), tumor differentiation grade stage (P = 0.005), JWA (*P* < 0.001) or XRCC1 (*P* < 0.001) mRNA expression in the whole cohort (Tables 2 and 3). Cox proportional hazard analysis revealed that high JWA (HR 0.22; 95% CI 0.13-0.37; *P* < 0.001) or high XRCC1 mRNA expression (HR 0.36; 95% CI 0.21-0.63; *P* < 0.001) emerged as independent prognostic factors associated with increased OS, whereas low tumor differentiation grade (HR 1.45; 95% CI 1.06-1.97; *P* = 0.019) emerged as higher risk for mortality associated with decreased mOS (Table 4).

**Table 4:** **Multivariate cox regression analysis of clinical characteristics and JWA, XRCC1 and BRCA1 expression associated with survival**

Clinical variables	HR	95%CI	*P* value
Gender (females *vs*. males)	0.83	0.56-1.22	0.349
ECOG, PS (0-1 *vs*. 2)	0.70	0.59-1.43	0.701
TNM stage (II *vs*. III or III *vs*. IV)	0.57	0.74-1.18	0.567
G stage (G1 *vs.* G2 or G2 *vs*. G3)	1.45	1.06-1.97	0.019
JWA (low *vs.* high)	0.22	0.13-0.37	<0.001
XRCC1 (low *vs.* high)	0.36	0.21-0.63	<0.001
BRCA1 (low *vs.* high)	1.14	0.80-1.62	0.485

HR = hazard ratios; y = years; G = differentiation grade; RR = response rate; CI = confidence interval.

## Discussion

This study was the first to detect the prognostic roles and synergistic effects of JWA/XRCC1/BRCA1 mRNA expression in paraffin-embedded tumor tissues on molecular staging for personalized therapy of advanced ESCC who received cisplatin- or docetaxel-based treatments.

In our study, JWA mRNA expression was significantly associated with patients’ gender in this cohort, which showed that median JWA expression level was higher in females than in males. In the previous studies, the alcohol and tobacco consumption which were found significantly higher in males than in females had the synergistic effects on the development of ESCC [30-32]. Both risk factors were proved to take part in the dysregulation of cell cycle, apoptosis and DNA repair [33-35]. JWA, as a DNA repair gene and anti-oncogene [24], might be correlated with the consumption of alcohol and tobacco in ESCC patients. In addition, estrogen can regulate transcription of genes associated with cell survival and proliferation by activating the estrogen receptor related pathways [36], which might explain the difference of JWA expression between males and females if JWA was involved in these pathways. However, the relationship between JWA and the alcohol/tobacco consumption or estrogen was not clear and need to be further studied. Also, we found that JWA expression level was positively correlated with tumor differentiation grade in ESCC patients. Though the loss of JWA expression has been discovered to inhibit the cell differentiation and cause more malignant phenotypes [8,23,37], it was still ambiguous whether JWA could be the important regulatory factor in differentiation-related pathways including JAK-STAT, Notch and Wnt signaling pathways [38-40]. Further studies should be carried out to discover whether and how JWA participated in these pathways.

In this study, high JWA or XRCC1 mRNA expression was correlated with longer overall survival in all the patients or in subgroups treated with different regimens and emerged as the independent prognostic factors for ESCC patients in this cohort. These findings were in agreement with the results in the gastric, hepatocellular and bladder carcinomas [23,24,41]. Increasing evidences implicated the role of JWA on oncogenic and metastatic phenotypes in several human cancers. Downregulation of JWA was found to be crucial for the invasion and metastasis of human tumor through elevated FAK expression, the induction of RhoA and MMP-2 activation [11,24,25]. In addition, JWA has significant predictive power for its correlation with tumor differentiation in the present study, which was known as an important factor for tumor progression. XRCC1 protein was deemed as a scaffold in the process of BER binding the DNA and recruiting other repair components after recognizing DNA breaks [14-19]. XRCC1 gene might be a valuable genetic marker for chemotherapy in various cancers as mentioned above. In present study, predictive roles of JWA and XRCC1 on survival with a similar trend were observed owing to the relationship between the two genes. JWA was found to cause XRCC1 transcription through increasing the affinity of E2F1 for binding to the XRCC1 promoter via MAPK signaling pathway and maintain the stability of the XRCC1 protein through inhibiting the ubiquitin-proteasome pathway [13]. We revealed that JWA alone was sufficient to predict the survival of advanced ESCC compared with combining JWA with XRCC1 according to ROC analysis, although this result was not consistent with the published data that combining JWA with XRCC1 had the synergistic effect on prognosis in gastric cancer [23].

DNA repair proteins were proved to play important roles in the process of repairing DNA damage caused by chemotherapeutics [5-7]. In this study, low expression of JWA mRNA was significantly correlated with increased mOS in patients treated with cisplatin-based regimens but not in those treated with docetaxel-based regimens though JWA was identified as microtubule-associated protein, which was consistent with the current evidence for the predictive value of JWA in resectable gastric cancer that underwent platinum-based adjuvant chemotherapy [23,25,42]. Also, JWA as a new BER protein was found to protect cells with induced DNA damage through upregulating XRCC1 and downregulating PARP-1, which might explain the predictive value of JWA to cisplatin sensitivity [13]. However, the predictive role of XRCC1 on the cisplatin sensitivity was not observed in this cohort. Seemingly, JWA mRNA expression was better than XRCC1 to be the prognostic factor for ESCC patients who received cisplatin-based treatment. Although previous researches had discovered some DNA repair proteins associated to the damage caused by radiation in vitro, we didn’t find effects of JWA on radiotherapy sensitivity in this study [43-45].

BRCA1 mRNA expression level was observed to be a valid predictor of inverse sensitivity to cisplatin or docetaxel in advanced ESCC, which was also shown in our previous study [7]. For effects of JWA or BRCA1 mRNA expression on survival of ESCC in terms with regimens, the synergistic effects of the two gene expression on cisplatin or docetaxel-based treatments were further explored. The patients with low JWA/low BRCA1 expressing benefited mostly from cisplatin-based treatment, and those with high JWA/high BRCA1 expression benefited mostly from docetaxel-based treatment. From the results, we can separate out the patients with low or high BRCA1 expression who could not benefit from cisplatin- or docetaxel-based treatment. Combining JWA with BRCA1 mRNA expression might be better than single gene expression currently used in personalized therapy for ESCC patients. It seems that combined NER and BER pathways are more significant to predict therapy outcome than single pathway [13,26].

Several limitations needed to be considered and clarified in this study, which included unplanned and retrospective analysis, lacking of validation group and relatively small number of patients in the subgroups. However, the prognostic and predictive roles of JWA/ XRCC1/BRCA1 mRNA expression were firstly proved in personalized therapy for patients with advanced ESCC treated with cisplain- or docetaxel-based regimens and indicated the need to further validate in prospective adequately designed clinical trials.

## Conclusions

In this study, we demonstrated that high JWA or XRCC1 mRNA expression emerged as the independent prognostic factors for ESCC patients and JWA alone was sufficient to predict the survival compared with combining JWA with XRCC1. Also, we further proved the synergistic effects of combining JWA with BRCA1 expression on cisplatin or docetaxel-based treatments. In summary, JWA, XRCC1 and BRCA1 mRNA expression as predictive markers could be used in molecular staging for personalized therapy in ESCC patients who received first-line cisplatin or docetaxel-based treatments.

## Additional files

Additional file 1: Table S1. Association of JWA, XRCC1 and BRCA1 expression with Median OS. **Table S2.** The AUC values of time-dependent ROC analyses. **Table S3.** Outcomes in different treatments according to JWA expression levels. **Table S4.** Outcomes in low or high JWA expression levels according to different treatments. **Table S5.** Outcomes in different treatments according to XRCC1 expression levels. **Table S6.** Outcomes in low or high XRCC1 expression levels according to different treatments. **Table S7.** Outcomes in different treatments according to BRCA1 expression levels. **Table S8.** Outcomes in low or high BRCA1 expression levels according to different treatments. **Table S9.** Outcomes of JWA and BRCA1 expression levels according to different treatments.
Additional file 2: Figure S1. Median OS according to JWA mRNA expression in different therapeutic subgroups. (A) Cisplatin/5-Fu-based chemotherapy. (B) Docetaxel/5-Fu-based chemotherapy. (C) Radiotherapy alone. (D) Cisplatin/5-Fu-based chemoradiotherapy. (E) Docetaxel/5-Fu-based chemoradiotherapy. **Figure S2.** Median OS according to XRCC1 mRNA expression in different therapeutic subgroups. (A) Cisplatin/5-Fu-based chemotherapy. (B) Docetaxel/5-Fu-based chemotherapy. (C) Radiotherapy alone. (D) Cisplatin/5-Fu-based chemoradiotherapy. (E) Docetaxel/5-Fu-based chemoradiotherapy. **Figure S3.** Median OS in low and high XRCC1 expression levels according to different therapeutic regimens. (A) Chemotherapy in low XRCC1 expression. (B) Chemoradiotherapy in low XRCC1 expression. (C) Chemotherapy in high XRCC1 expression. (D) Chemoradiotherapy in high XRCC1 expression. **Figure S4.** Median OS according to BRCA1 mRNA expression in different therapeutic subgroups. (A) Cisplatin/5-Fu-based chemotherapy. (B) Docetaxel/5-Fu-based chemotherapy. (C) Radiotherapy alone. (D) Cisplatin/5-Fu-based chemoradiotherapy. (E) Docetaxel/5-Fu-based chemoradiotherapy. **Figure S5.** Median OS in low and high BRCA1 expression levels according to different therapeutic regimens. (A) Chemotherapy in low BRCA1 expression. (B) Chemoradiotherapy in low BRCA1 expression. (C) Chemotherapy in high BRCA1 expression. (D) Chemoradiotherapy in high BRCA1 expression.
