# Supplementary material for: Effects of JWA, XRCC1 and BRCA1 mRNA expression on molecular staging for personalized therapy in patients with advanced esophageal squamous cell carcinoma
Source: BMC Cancer. 2015 Apr 30;15:331. doi: 10.1186/s12885-015-1364-0 (PMC4469327; doi:10.1186/s12885-015-1364-0)
Supplement: Additional file 1: Table S1. — Association of JWA, XRCC1 and BRCA1 expression with Median OS. Table S2. The AUC values of time-dependent ROC analyses. Table S3. Outcomes in different treatments according to JWA expression levels. Table S4. Outcomes in low or high JWA expression levels according to different treatments. Table S5. Outcomes in different treatments according to XRCC1 expression levels. Table S6. Outcomes in low or high XRCC1 expression levels according to different treatments. Table S7. Outcomes in different treatments according to BRCA1 expression levels. Table S8. Outcomes in low or high BRCA1 expression levels according to different treatments. Table S9. Outcomes of JWA and BRCA1 expression levels according to different treatments. [file 12885_2015_1364_MOESM1_ESM.doc]

**Additional file 1**

**Table S1** Association of JWA, XRCC1 and BRCA1 expression with median OS.

| **Genes** |  | **mOS** |  |
| --- | --- | --- | --- |
| **JWA and XRCC1** | **No.** | **Median(95%CI)** | ***P* value** |
| JWA low/ XRCC1 low | 62 | 8.0(6.8-9.2) | 1 |
| JWA low/ XRCC1 high | 11 | 10.0(6.8-11.1) | 0.112 |
| JWA high/ XRCC1 low | 10 | 17.0(16.0-18.0) | <0.001 |
| JWA high/ XRCC1 high | 62 | 21.0(16.4-24.6) | <0.001 |

mOS = median overall survival; CI = confidence interval.

**Table S2** The AUC values of time-dependent ROC analyse**s.**

|  | **AUC values** | | | | | | | |
| --- | --- | --- | --- | --- | --- | --- | --- | --- |
| **Time (year)** | **One** | | **Two** | | **Three** | | **Four** | |
|  | **95%CI** | ***P*** | **95%CI** | ***P*** | **95%CI** | ***P*** | **95%CI** | ***P*** |
| **Variables** | 0.651  (0.515-0.788) | 0.043 | 0.54  (0.385-0.699) | 0.574 | 0.651  (0.515-0.788) | 0.043 | 0.489  (0.330-0.648) | 0.880 |
| **XRCC1/ variable** | 0.750  (0.638-0.862) | 0.001 | 0.677  (0.564-0.808) | 0.018 | 0.750  (0.638-0.862) | 0.001 | 0.672  (0.542-0.802) | 0.022 |
| **JWA/ variable** | 0.769  (0.655-0.882) | <0.001 | 0.702  (0.605-0.799) | 0.007 | 0.769  (0.655-0.882) | <0.001 | 0.702  (0.605-0.799) | 0.007 |
| **JWA/ XRCC1/ variable** | 0.773  (0.667-0.878) | <0.001 | 0.718  (0.620-0.817) | 0.004 | 0.765  (0.657-0.874) | <0.001 | 0.656  (0.517-0.795) | 0.037 |

.

AUC = area under the curve; variable = TNM stage and G stage.

**Table S3** Outcomes in different treatments according to JWA expression levels.

|  | | **RR, No. (%)** | | | **mOS** | | |
| --- | --- | --- | --- | --- | --- | --- | --- |
| **Treatment** | **JWA** | **CR+PR** | **SD+PD** | ***P* value** | **No.** | **Median(95%CI)** | ***P* value** |
| Cis/5-Fu | Low | 57.1 | 42.9 |  | 14 | 8.0(7.1-8.9) |  |
|  | High | 71.4 | 28.6 | 0.383 | 21 | 19.0(16.1-21.9) | <0.001 |
| Doc/5-Fu | Low | 64.0 | 36.0 |  | 25 | 8.0(5.6-10.4) |  |
|  | High | 84.6 | 15.4 | 0.268 | 13 | 18.0(14.5-21.5) | <0.001 |
| Radiotherapy alone | Low | 75 | 25 |  | 4 | 7.5(4.6-10.4) |  |
|  | high | 100 | 0 | 0.14 | 8 | 20(12.4-27.6) | <0.001 |
| Radiotherapy+ Cis/5-Fu | Low | 91.7 | 8.3 |  | 12 | 11.0(9.9-12.2) |  |
|  | High | 83.3 | 16.7 | 0.511 | 18 | 23.0(12.2-33.8) | 0.009 |
| Radiotherapy+Doc/5-Fu | low | 61.1 | 38.9 |  | 18 | 7.0(6.2-7.8) |  |
|  | High | 91.7 | 8.3 | 0.064 | 12 | 23.0(13.9-32.1) | <0.001 |

Cis = cisplatin; 5-Fu = 5-fluorouracil; Doc = docetaxel; CR = complete response; PR = partial response; SD = stable disease; PD = progress disease; RR = response rate; mOS = median overall survival; CI = confidence interval.

**Table S4** Outcomes in low or high JWA expression levels according to different treatments.

|  |  | **RR, No. (%)** | | | **mOS** | | |
| --- | --- | --- | --- | --- | --- | --- | --- |
| **JWA** | **Treatment** | **CR+PR** | **SD+PD** | ***P* value** | **No.** | **Median(95%CI)** | ***P* value** |
| Low | Cis/5-Fu | 57.1 | 42.9 |  | 14 | 10.0(6.4-13.6) |  |
|  | Doc/5-Fu | 64 | 36 | 0.673 | 25 | 8.0(6.2-9.8) | 0.015 |
| Low | Radiotherapy + Cis/5-Fu | 91.7 | 8.3 |  | 12 | 11.0(9.9-12.1) |  |
|  | Radiotherapy + Doc/5-Fu | 67.7 | 33.3 | 0.286 | 18 | 7.0(6.2-7.8) | 0.001 |
|  | Radiotherapy alone | 75 | 25 |  | 4 | 7.5(4.6-10.4) | 0.537 |
| High | Cis/5-Fu | 71.4 | 28.6 |  | 21 | 19.0(16.1-21.9) |  |
|  | Doc/5-Fu | 84.6 | 15.6 | 0.378 | 13 | 18.0(14.5-21.5) | 0.783 |
| High | Radiotherapy+ Cis/5-Fu | 83.3 | 16.7 |  | 18 | 23.0(12.2-33.8) |  |
|  | Radiotherapy + Doc/5-Fu | 91.7 | 8.3 | 0.423 | 12 | 23.0(13.9-32.1) | 0.88 |
|  | Radiotherapy alone | 100 | 0 |  | 8 | 20.0(12.4-27.6) | 0.505 |

Cis = cisplatin; 5-Fu = 5-fluorouracil; Doc = docetaxel; CR = complete response; PR = partial response; SD = stable disease; PD = progress disease; RR = response rate; mOS = median overall survival; CI = confidence interval.

**Table S5** Outcomes in different treatments according to XRCC1 expression levels.

|  | | **RR, No. (%)** | | | **mOS** | | |
| --- | --- | --- | --- | --- | --- | --- | --- |
| **Treatment** | **XRCC1** | **CR+PR** | **SD+PD** | ***P* value** | **No.** | **Median (95%CI)** | ***P* value** |
| Cis/5-Fu | Low | 38.5 | 61.5 |  | 13 | 9.0(5.4-12.5) |  |
|  | High | 81.8 | 18.2 | 0.06 | 22 | 19.0(16.1-21.9) | <0.001 |
| Doc/5-Fu | Low | 62.5 | 37.5 |  | 24 | 7.0(4.1-10.0) |  |
|  | High | 85.7 | 14.3 | 0.16 | 14 | 17.0(13.3-20.7) | <0.001 |
| Radiotherapy alone | Low | 80 | 20 |  | 5 | 10.0(5.7-14.3) |  |
|  | high | 100 | 0 | 0.217 | 7 | 21.0(8.2-33.8) | 0.009 |
| Radiotherapy+Cis/5-Fu | Low | 92.3 | 7.7 |  | 13 | 12.0(8.5-15.5) |  |
|  | High | 82.3 | 17.7 | 0.427 | 17 | 23.0(15.5-30.5) | 0.004 |
| Radiotherapy+Doc/5-Fu | low | 77.8 | 22.2 |  | 18 | 7.0(5.6-8.4) |  |
|  | High | 75 | 25 | 0.86 | 12 | 23.0(9.3-36.7) | <0.001 |

Cis = cisplatin; 5-Fu = 5-fluorouracil; Doc = docetaxel; CR = complete response; PR = partial response; SD = stable disease; PD = progress disease; RR = response rate; mOS = median overall survival; CI = confidence interval.

**Table S6** Outcomes in low or high XRCC1 expression levels according to different treatments.

|  | | **RR, No. (%)** | | | **mOS** | | |
| --- | --- | --- | --- | --- | --- | --- | --- |
| **XRCC1** | **Treatment** | **CR+PR** | **SD+PD** | ***P* value** | **No.** | **Median(95%CI)** | ***P* value** |
| Low | Cis/5-Fu | 38.5 | 61.5 |  | 13 | 9.0(5.5-12.5) |  |
|  | Doc/5-Fu | 62.5 | 37.5 | 0.188 | 24 | 7.0(4.1-9.9) | 0.142 |
| Low | Radiotherapy + Cis/5-Fu | 92.3 | 7.7 |  | 13 | 12.0(7.8-16.2) |  |
|  | Radiotherapy + Doc/5-Fu | 77.8 | 22.2 | 0.487 | 18 | 8.0(5.9-10.1) | 0.055 |
|  | Radiotherapy alone | 80 | 20 |  | 5 | 9.0(4.7.2-13.3) | 0.418 |
| High | Cis/5-Fu | 81.8 | 18.2 |  | 22 | 19.0(16.1-21.9) |  |
|  | Doc/5-Fu | 85.7 | 14.3 | 0.76 | 14 | 17.0(13.3-20.7) | 0.781 |
| High | Radiotherapy + Cis/5-Fu | 82.3 | 17.7 |  | 17 | 23.0(15.5-30.5) |  |
|  | Radiotherapy + Doc/5-Fu | 75.0 | 25.0 | 0.325 | 12 | 23.0(9.3-36.3) | 0.878 |
|  | Radiotherapy alone | 100 | 0 |  | 7 | 21.0(8.2-33.8) | 0.356 |

Cis = cisplatin; 5-Fu = 5-fluorouracil; Doc = docetaxel; CR = complete response; PR = partial response; SD = stable disease; PD = progress disease; RR = response rate; mOS = median overall survival; CI = confidence interval.

**Table S7** Outcomes in different treatments according to BRCA1 expression levels.

|  | | **RR, No. (%)** | | | **mOS** | | |
| --- | --- | --- | --- | --- | --- | --- | --- |
| **Treatment** | **BRCA1** | **CR+PR** | **SD+PD** | ***P*value** | **No.** | **Median (95%CI)** | ***P* value** |
| Cis/5-Fu | Low | 75.0 | 25.0 |  | 20 | 18.0(15.5-20.5) |  |
|  | High | 73.3 | 26.7 | 1.000 | 15 | 8.0(6.5-9.5) | 0.002 |
| Doc/5-Fu | Low | 80 | 20 |  | 20 | 8.0(5.7-10.3) |  |
|  | High | 66.7 | 33.3 | 0.351 | 18 | 13.0(10.3-15.7) | 0.011 |
| Radiotherapy alone | Low | 71.4 | 28.6 |  | 7 | 15.0(4.7-25.3) |  |
|  | High | 100 | 0 | 0.19 | 5 | 20.0(7.1-32.9) | 0.221 |
| Radiotherapy+Cis/5-Fu | Low | 76.5 | 23.5 |  | 17 | 21.0(13.4-28.6) |  |
|  | High | 76.9 | 23.1 | 0.977 | 13 | 12.0(9.7-14.3) | <0.001 |
| Radiotherapy+Doc/5-Fu | Low | 80 | 20 |  | 10 | 7.0(5.7-8.2) |  |
|  | High | 75 | 25 | 0.248 | 20 | 14.0(11.4-16.6) | 0.037 |

Cis = cisplatin; 5-Fu = 5-fluorouracil; Doc = docetaxel; CR = complete response; PR = partial response; SD = stable disease; PD = progress disease; RR = response rate; mOS = median overall survival; CI = confidence interval.

**Table S8** Outcomes in low or high BRCA1 expression levels according to different treatments.

|  | | **RR, No. (%)** | | | **mOS** | | |
| --- | --- | --- | --- | --- | --- | --- | --- |
| **BRCA1** | **Treatment** | **CR+PR** | **SD+PD** | ***P* value** | **No.** | **Median(95%CI)** | ***P* value** |
| Low | Cis/5-Fu | 75 | 25 |  | 20 | 18.0(15.5-20.5) |  |
|  | Doc/5-Fu | 80 | 20 | 0.705 | 20 | 8.0(5.7-10.3) | 0.001 |
| Low | Radiotherapy + Cis/5-Fu | 76.9 | 23.1 |  | 17 | 21.0(13.4-28.6) |  |
|  | Radiotherapy + Doc/5-Fu | 80 | 20 | 0.919 | 10 | 7.0(5.7-8.3) | 0.003 |
|  | Radiotherapy alone | 71.4 | 28.6 |  | 7 | 15.0(4.7-25.3) | 0.16 |
| High | Cis/5-Fu | 73.3 | 26.7 |  | 15 | 8.0(6.5-9.5) |  |
|  | Doc/5-Fu | 66.7 | 33.3 | 0.722 | 18 | 13.0(10.3-15.7) | 0.02 |
| High | Radiotherapy + Cis/5-Fu | 69.2 | 30.8 |  | 13 | 12.0(9.7-14.3) |  |
|  | Radiotherapy + Doc/5-Fu | 75 | 25 | 0.523 | 20 | 14.0(11.4-16.6) | 0.048 |
|  | Radiotherapy alone | 100 | 0 |  | 5 | 20.0(7.1-32.9) | 0.034 |

Cis = cisplatin; 5-Fu = 5-fluorouracil; Doc = docetaxel; CR = complete response; PR = partial response; SD = stable disease; PD = progress disease; RR = response rate; mOS = median overall survival; CI = confidence interval.

**Table S9** Outcomes of JWA and BRCA1 expression levels according to different treatments.

|  | | **RR, No. (%)** | | | **mOS** | | |
| --- | --- | --- | --- | --- | --- | --- | --- |
| **JWA and BRCA1** | **Treatment** | **CR+PR** | **SD+PD** | ***P* value** | **No.** | **Median(95%CI)** | ***P* value** |
| JWA low/ BRCA1 low | Cis-based | 71.4 | 28.6 |  | 14 | 20.0(16.3-23.7) |  |
|  | Doc-based | 73.7 | 26.3 | 0.886 | 19 | 8.0(6.0-10.0) | <0.001 |
| JWA low/ BRCA1high | Cis-based | 70.6 | 29.4 |  | 17 | 9.0(5.0-13.0) |  |
|  | Doc-based | 52.9 | 47.1 | 0.29 | 17 | 10.0(7.6-12.4) | 0.22 |
| JWA high/ BRCA1 low | Cis-based | 77.3 | 22.7 |  | 22 | 18.0(14.7-21.3) |  |
|  | Doc-based | 100 | 0 | 0.086 | 11 | 7.0(5.8-8.2) | 0.09 |
| JWA high/ BRCA1high | Cis-based | 81.8 | 18.2 |  | 11 | 12.0(9.1-14.9) |  |
|  | Doc-based | 81.8 | 18.2 | 0.671 | 22 | 18.0(9.8-26.2) | 0.044 |

Cis = cisplatin; 5-Fu = 5-fluorouracil; Doc = docetaxel; CR = complete response; PR = partial response; SD = stable disease; PD = progress disease; RR = response rate; mOS = median overall survival; CI = confidence interval.
