# Supplementary material for: Effects of JWA, XRCC1 and BRCA1 mRNA expression on molecular staging for personalized therapy in patients with advanced esophageal squamous cell carcinoma
Source: BMC Cancer. 2015 Apr 30;15:331. doi: 10.1186/s12885-015-1364-0 (PMC4469327; doi:10.1186/s12885-015-1364-0)
Supplement: Additional file 2: Figure S1. — Median OS according to JWA mRNA expression in different therapeutic subgroups. (A) Cisplatin/5-Fu-based chemotherapy. (B) Docetaxel/5-Fu-based chemotherapy. (C) Radiotherapy alone. (D) Cisplatin/5-Fu-based chemoradiotherapy. (E) Docetaxel/5-Fu-based chemoradiotherapy. Figure S2. Median OS according to XRCC1 mRNA expression in different therapeutic subgroups. (A) Cisplatin/5-Fu-based chemotherapy. (B) Docetaxel/5-Fu-based chemotherapy. (C) Radiotherapy alone. (D) Cisplatin/5-Fu-based chemoradiotherapy. (E) Docetaxel/5-Fu-based chemoradiotherapy. Figure S3. Median OS in low and high XRCC1 expression levels according to different therapeutic regimens. (A) Chemotherapy in low XRCC1 expression. (B) Chemoradiotherapy in low XRCC1 expression. (C) Chemotherapy in high XRCC1 expression. (D) Chemoradiotherapy in high XRCC1 expression. Figure S4. Median OS according to BRCA1 mRNA expression in different therapeutic subgroups. (A) Cisplatin/5-Fu-based chemotherapy. (B) Docetaxel/5-Fu-based chemotherapy. (C) Radiotherapy alone. (D) Cisplatin/5-Fu-based chemoradiotherapy. (E) Docetaxel/5-Fu-based chemoradiotherapy. Figure S5. Median OS in low and high BRCA1 expression levels according to different therapeutic regimens. (A) Chemotherapy in low BRCA1 expression. (B) Chemoradiotherapy in low BRCA1 expression. (C) Chemotherapy in high BRCA1 expression. (D) Chemoradiotherapy in high BRCA1 expression. [file 12885_2015_1364_MOESM2_ESM.pdf]

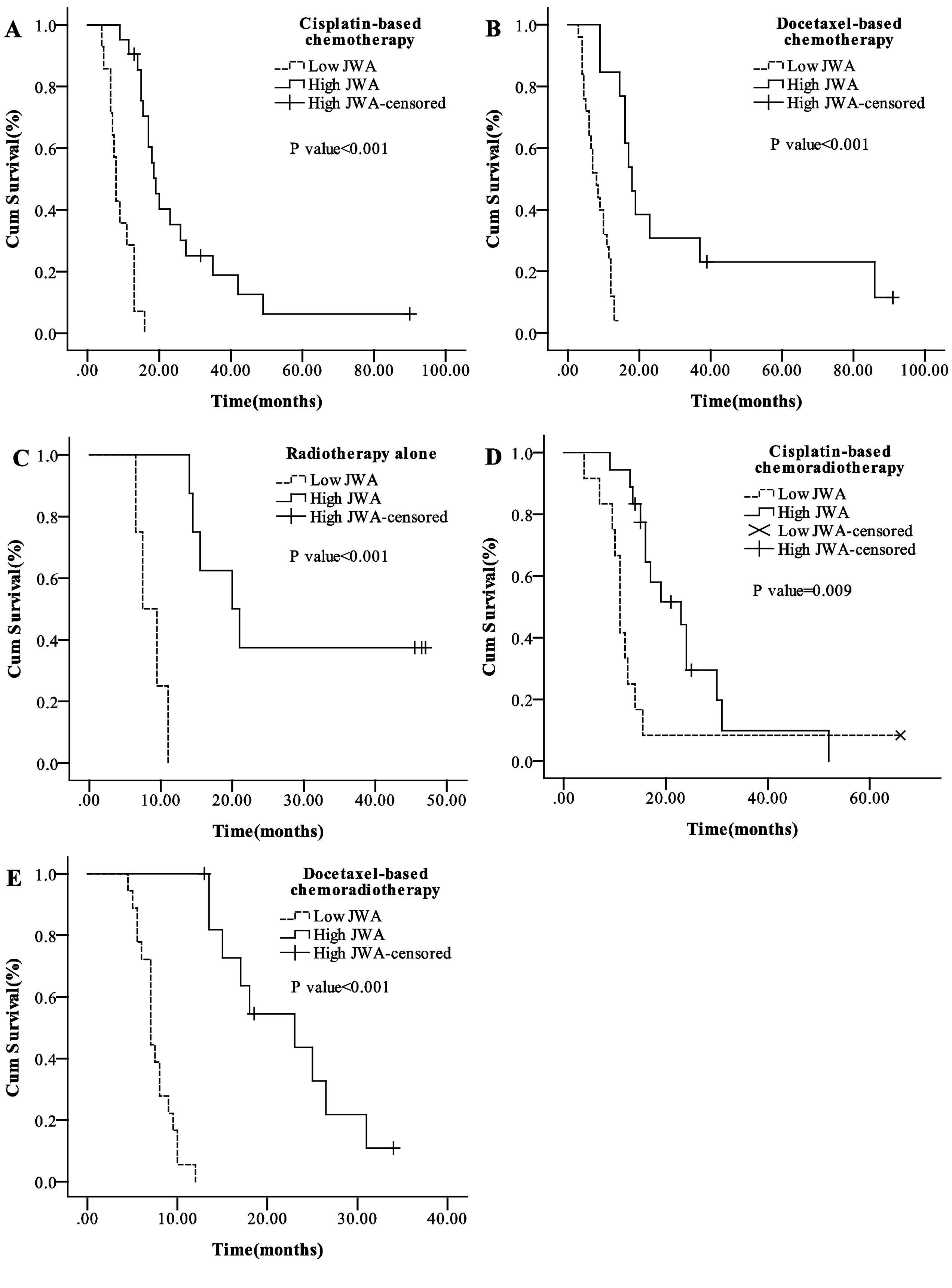

Additional file 1: Figure S1 Median OS according to JWA mRNA expression in different therapeutic subgroups. (A) Cisplatin/5-Fu-based chemotherapy. (B) Docetaxel/5-Fu-based chemotherapy. (C) Radiotherapy alone. (D) Cisplatin/5-Fu-based chemoradiotherapy. (E) Docetaxel/5-Fu-based chemoradiotherapy.

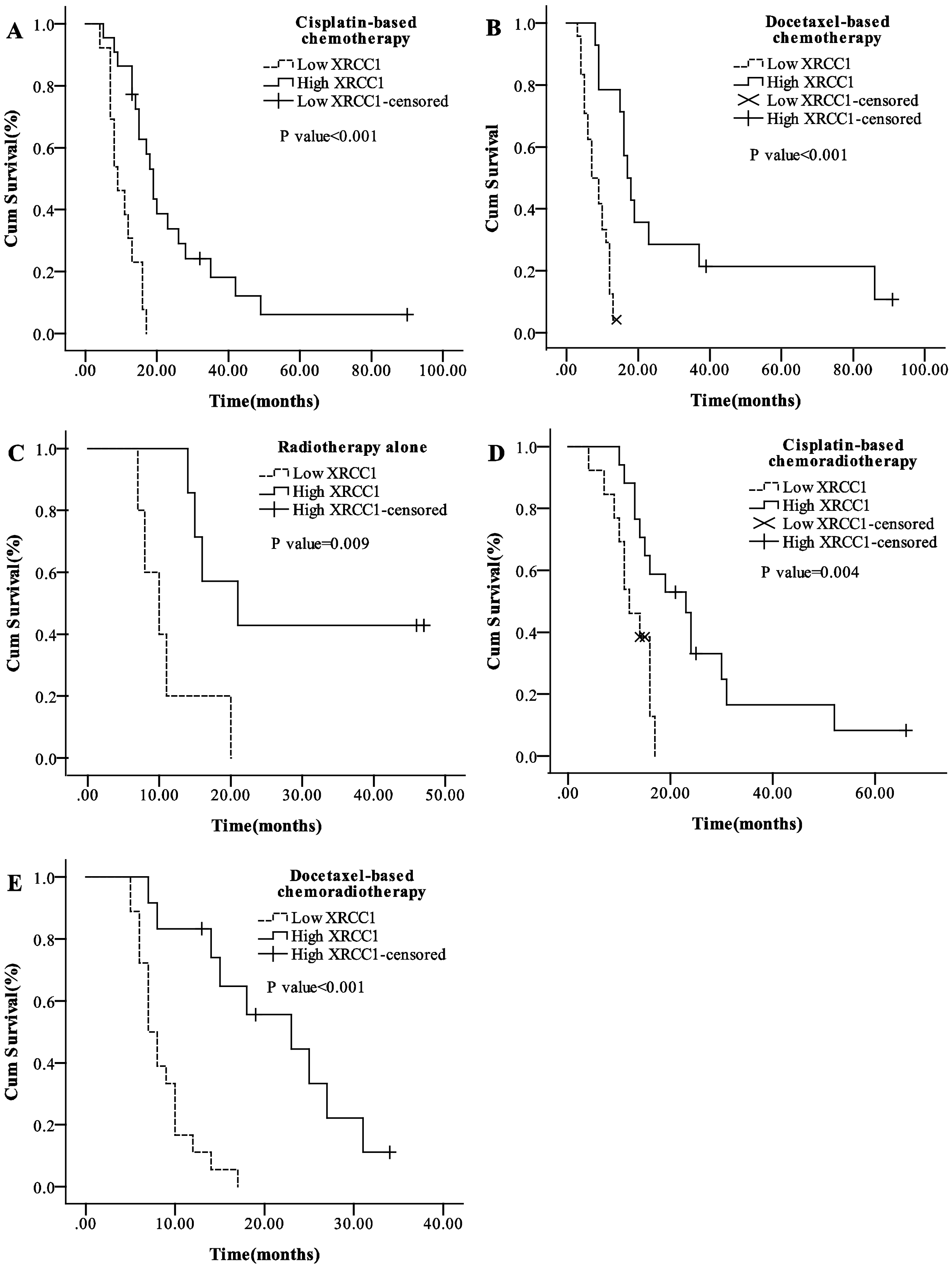

Additional file 1: Figure S2 Median OS according to XRCC1 mRNA expression in different therapeutic subgroups. (A) Cisplatin/5-Fu-based chemotherapy. (B) Docetaxel/5-Fu-based chemotherapy. (C) Radiotherapy alone. (D) Cisplatin/5-Fu-based chemoradiotherapy. (E) Docetaxel/5-Fu-based chemoradiotherapy.

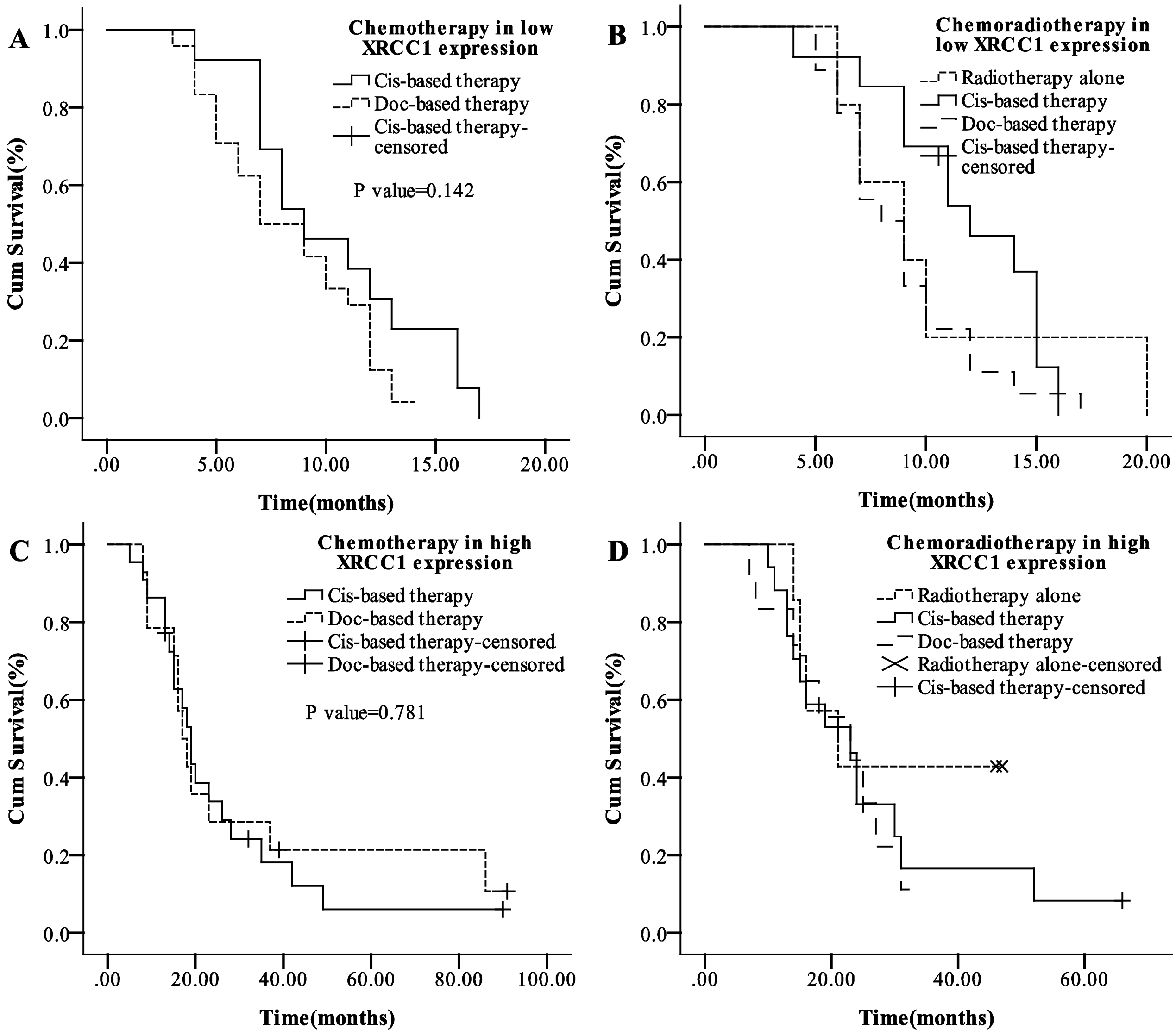

Additional file 1: Figure S3 Median OS in low and high XRCC1 expression levels according to different therapeutic regimens. (A) Chemotherapy in low XRCC1 expression. (B) Chemoradiotherapy in low XRCC1 expression. (C) Chemotherapy in high XRCC1 expression. (D) Chemoradiotherapy in high XRCC1 expression.

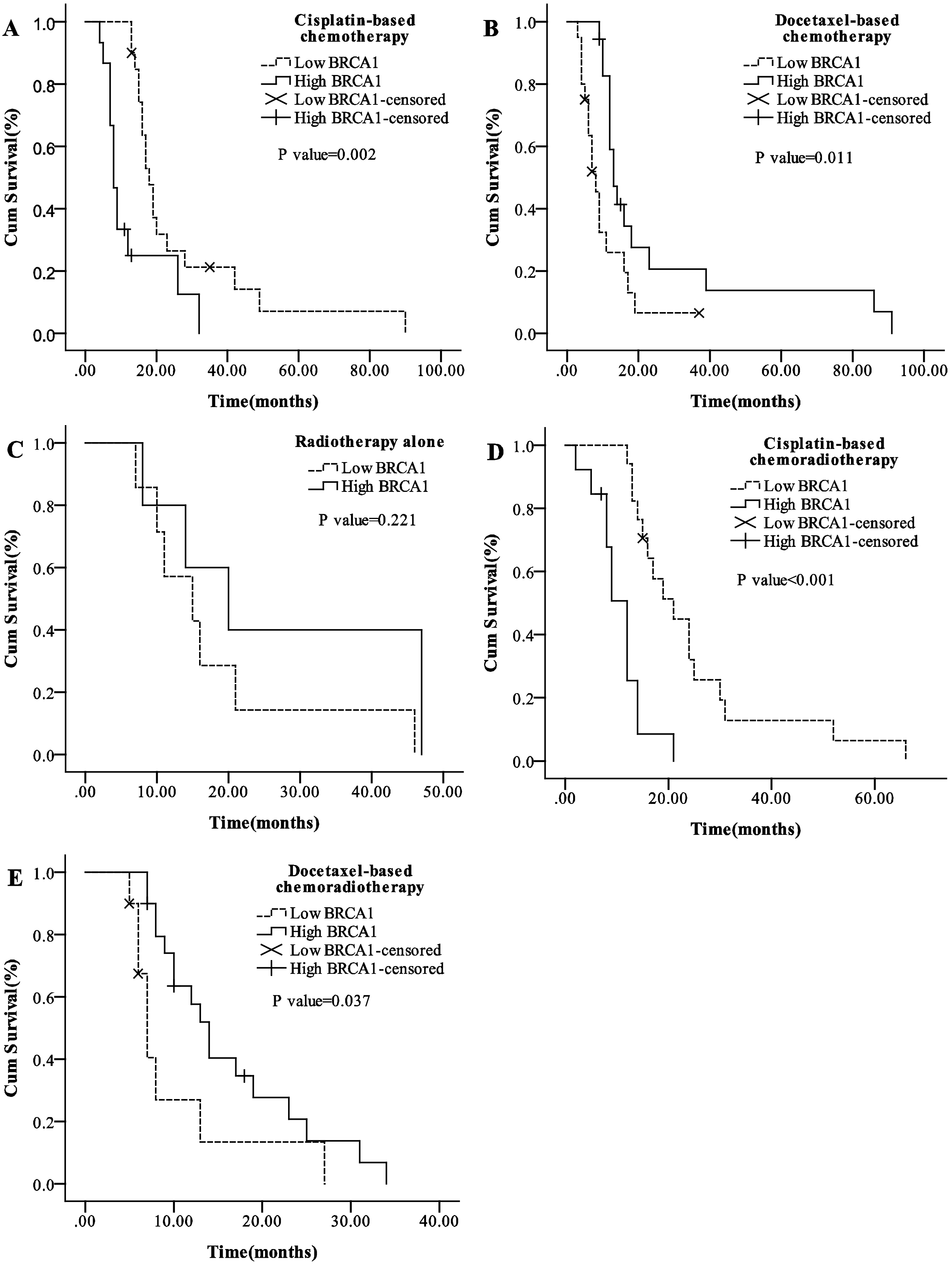

Additional file 1: Figure S4 Median OS according to BRCA1 mRNA expression in different therapeutic subgroups. (A) Cisplatin/5-Fu-based chemotherapy. (B) Docetaxel/5-Fu-based chemotherapy. (C) Radiotherapy alone. (D) Cisplatin/5-Fu-based chemoradiotherapy. (E) Docetaxel/5-Fu-based chemoradiotherapy.

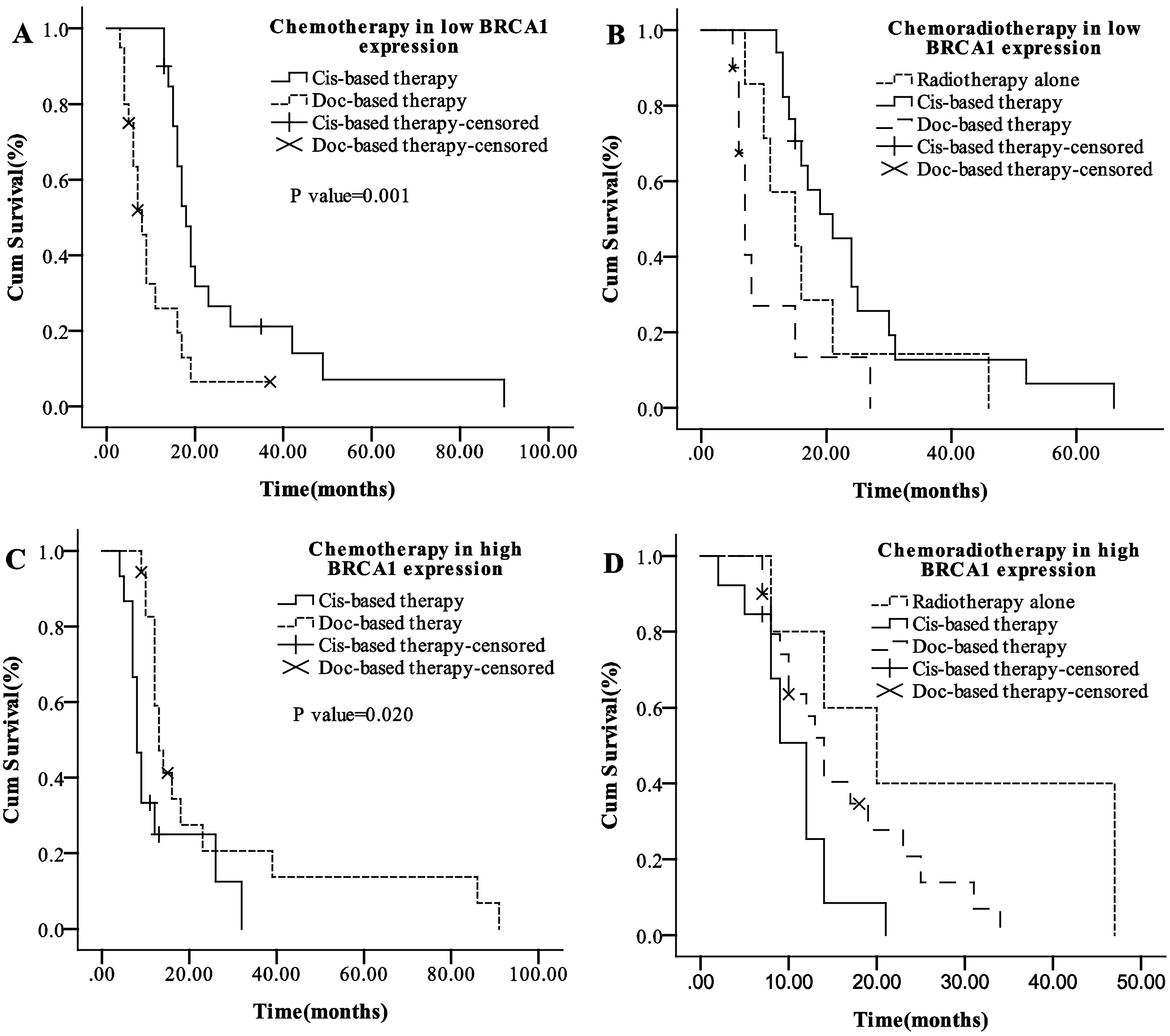

Additional file 1: Figure S5 Median OS in low and high BRCA1 expression levels according to different therapeutic regimens. (A) Chemotherapy in low BRCA1 expression. (B) Chemoradiotherapy in low BRCA1 expression. (C) Chemotherapy in high BRCA1 expression. (D) Chemoradiotherapy in high BRCA1 expression.
